# Anti-glycin-receptor antibody related stiff-person syndrome under treatment with an immune checkpoint inhibitor

**DOI:** 10.1007/s00415-020-10351-2

**Published:** 2020-12-24

**Authors:** Nils Schröter, Cornelius Weiller, Sebastian Rauer, Cornelius F. Waller

**Affiliations:** 1 Clinic of Neurology and Neurophysiology, Medical Center, Faculty of Medicine, University of Freiburg, Breisacher Str. 64, 79106 Freiburg, Germany; 2 Department of Haematology, Oncology and Stem Cell Transplantation, Faculty of Medicine, University Medical Centre Freiburg, University of Freiburg, Freiburg, Germany

Dear Sirs,

We here report the case of a 59-year-old male patient who developed a stiff-person syndrome (SPS) as a possibly immune-related adverse event after receiving Pembrolizumab as first-line therapy of adenocarcinoma of the lung.

In September 2019, a 59-year-old male patient presented to our emergency department with reoccurring painful, immobilizing muscle spasms, stiffness, and falls which had lasted for approximately 10 days. Spasms were triggered by any voluntary movement, sensory, emotional or acoustic stimuli and were located mainly in the legs and the lower back, leading to a lumbar hyperlordosis. The patient reported an improvement of spasm severity and frequency under self-medication with cannabidiol and baclofen. He suffered from stage IV adenocarcinoma of the lung, treated with the anti-PD-1 antibody pembrolizumab. The patient had suffered from pembrolizumab associated increase in transaminases and diarrhea, both grade I, in August 2019 dissolving without further intervention.

On physical examination, the patient presented with hyperreflexia of the lower extremities with spastic tendencies but no weakness or other neurological deficit. Magnet resonance imaging (MRI) of the head and spine were unremarkable. Analysis of cerebrospinal fluid (CSF) revealed mild pleocytosis, 7/µl (< 5), and mild disturbances of the blood–brain–barrier with a CSF/serum quotient of albumin of 9.4 (< 8). Initial analysis of paraneoplastic anti-neuronal antibodies and autoimmune encephalitis antibodies, including anti-GAD, anti-amphysisin, anti-DPPX, and anti-GABA-B, were negative in CSF and blood serum, respectively. A secondary analysis revealed anti-glycine receptor antibodies (anti-GlyR). Pathological evaluation and immunophenotypical analysis of cells were unremarkable. Repeated staging by CT-thorax and abdomen did not exhibit new tumor growth. In the synopsis of anamnesis, clinical findings and data of laboratory testing the clinical diagnosis of anti-GlyR related SPS was made.

We initiated therapy with IV immunoglobulin (IVIG) for 5 days and symptomatic therapy with diazepam and morphine stopping symptom progression. Due to reoccurring muscle spasms, the patient was still immobilized and highly impaired in quality of life. In close agreement with the treating oncologists, we followed up IVIG therapy with methylprednisolone 40 mg per day. This led to an improvement in muscle spasm frequency and severity (Fig. 1).

**Fig. 1:**
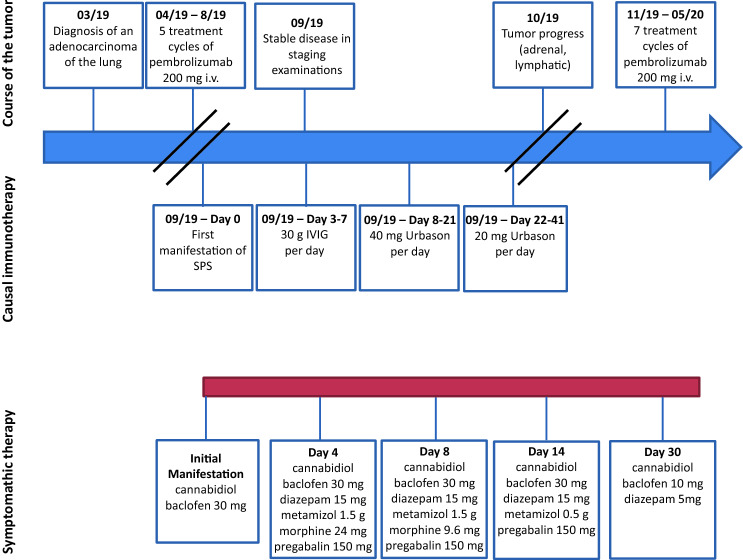
Timeline of the case reported. The upper line shows the course of the tumor and the pembrolizumab therapy. The middle row shows the course of causal therapy for stiff-person syndrome. The bottom line shows the course of the symptomatic therapy of the stiff-person syndrome. SPS, stiff-person syndrome; IVIG, intravenous immunoglobulin therapy

After 4 weeks of rehabilitation, symptoms were alleviated. Even though the patient still suffered from reoccurring muscle spasms and stiffness he was able to walk 500 m using a rollator. In October 2019, 3 months after the last infusion of pembrolizumab, tumor progress was detected and treatment with pembrolizumab was reinitiated. After a follow up of six months after initial presentation no relapse of SPS occurred and ^18^F-FDG PET showed stabilization of tumor manifestations.

We present the first case of a stiff-person syndrome as an immune-related adverse event (irAE) under checkpoint-inhibitors. The diagnosis of anti-GlyR positive SPS was based on the positive antibody results, the classic phenotype comprising co-contraction of agonists and antagonists, rigidity, lumbar hyperlordosis and “startle” induced spasms by tactile, acoustic or emotional triggers [1]. This assumption is supported by the apparent response to immunoglobulins and steroids.

The PD-1 receptor plays a key role in preventing T-cell mediated autoimmune responses [2]. That is why pharmacologically inhibiting the function of the PD-1 pathway leads on the one hand in PD-1 expressing malignancies to enhanced antitumor immune response and on the other hand, predisposes to irAEs that can occur in up to 27% of patients. Typically irAEs manifest in the skin, endocrine system, gastrointestinal tract or liver.

SPS can occur as a rare paraneoplastic syndrome in patients without treatment with ICIs [3]. In our view, the lack of tumor progression in the staging examinations and the long latency between tumor growth and the onset of SPS indicates a non-paraneoplastic genesis.

Similar to previous irAEs, SPS remained stable under the continuation of pembrolizumab. This is in line with current ASCO clinical practice guidelines regarding other neurological adverse events, stating that ICIs can be resumed in close consultation with the patient after symptoms have declined [4].

According to the present case report, SPS might be a rare but important irAE checkpoint inhibitors clinicians should be aware of.

## References

[CR1] Baizabal-Carvallo JF, Jankovic J (2015) Stiff-person syndrome: insights into a complex autoimmune disorder. J Neurol Neurosurg Psychiatry [Internet]. 86(8):840–8. Available from: https://jnnp.bmj.com/content/86/8/840 10.1136/jnnp-2014-309201 25511790

[CR2] Francisco LM, Sage PT, Sharpe AH (2010) The PD-1 pathway in tolerance and autoimmunity. Immunol Rev [Internet]. 236:219. Available from: https://www.ncbi.nlm.nih.gov/pmc/articles/PMC2919275/10.1111/j.1600-065X.2010.00923.x PMC2919275 20636820

[CR3] El-Abassi R, Soliman MY, Villemarette-Pittman N, England JD (2019) SPS: Understanding the complexity. J Neurol Sci [Internet]. 404:137–49. Available from: http://www.sciencedirect.com/science/article/pii/S0022510X19302813 10.1016/j.jns.2019.06.021 31377632

[CR4] Brahmer JR, Lacchetti C, Schneider BJ, Atkins MB, Brassil KJ, Caterino JM, et al (2018) Management of immune-related adverse events in patients treated with immune checkpoint inhibitor therapy: American Society of Clinical Oncology Clinical Practice Guideline. J Clin Oncol Off J Am Soc Clin Oncol [Internet]. 36(17):1714–68. Available from: https://www.ncbi.nlm.nih.gov/pmc/articles/PMC6481621/10.1200/JCO.2017.77.6385 PMC6481621 29442540

